# Chemical activation of a food deprivation signal extends lifespan

**DOI:** 10.1111/acel.12492

**Published:** 2016-05-24

**Authors:** Mark Lucanic, Theo Garrett, Ivan Yu, Fernando Calahorro, Azar Asadi Shahmirzadi, Aaron Miller, Matthew S. Gill, Robert E. Hughes, Lindy Holden‐Dye, Gordon J. Lithgow

**Affiliations:** ^1^ Buck Institute for Research on Aging8001 Redwood BoulevardNovatoCAUSA; ^2^ Dominican University of California50 Acacia AvenueSan RafaelCAUSA; ^3^ Center for Biological SciencesInstitute for Life SciencesUniversity of SouthamptonSouthamptonUK; ^4^ Davis School of GerontologyUniversity of Southern CaliforniaLos AngelesCAUSA; ^5^ Department of Metabolism & AgingThe Scripps Research Institute‐Scripps Florida130 Scripps WayJupiterFL33458

**Keywords:** Aging, Pharmacogenetics, Drug Discovery, Caenorhabditis, dietary restriction

## Abstract

Model organisms subject to dietary restriction (DR) generally live longer. Accompanying this lifespan extension are improvements in overall health, based on multiple metrics. This indicates that pharmacological treatments that mimic the effects of DR could improve health in humans. To find new chemical structures that extend lifespan, we screened 30 000 synthetic, diverse drug‐like chemicals in *Caenorhabditis elegans* and identified several structurally related compounds that acted through DR mechanisms. The most potent of these NP1 impinges upon a food perception pathway by promoting glutamate signaling in the pharynx. This results in the overriding of a GPCR pathway involved in the perception of food and which normally acts to decrease glutamate signals. Our results describe the activation of a dietary restriction response through the pharmacological masking of a novel sensory pathway that signals the presence of food. This suggests that primary sensory pathways may represent novel targets for human pharmacology.

## Introduction

Aging is profoundly influenced by environmental factors such as temperature, toxins, and nutrient availability. Sensory systems that detect environmental stimulus mediate at least part of this influence on lifespan. In the nematode *C. elegans,* as well as in *D. melanogaster,* the removal of sensory neurons can modulate the lifespan of the organism (Apfeld & Kenyon, 1999; Alcedo & Kenyon, 2004; Libert *et al*., 2007; Poon *et al*., 2010; Ostojic *et al*., 2014). Recent work has also shown that sensory signaling can also modulate the lifespan of vertebrates (Riera *et al*., 2014).

Progressive dysfunction and the breakdown of molecular pathways that control homeostatic processes such as energy metabolism, protein quality, and organismal growth have all been implicated in aging and promoting the integrity of these systems can delay aging (Taylor & Dillin, 2011; Sahin & DePinho, 2012; Shore & Ruvkun, 2013). Alterations in sensory neuron function can influence these processes in non‐neuronal tissues. For example, in the nematode *Caenorhabditis elegans*, the activity of the AFD neurons is required for the induction of heat‐shock genes in multiple non‐neuronal tissues, demonstrating that a stress response critical in promoting protein homeostasis (proteostasis) is under cell nonautonomous neuronal control (Prahlad *et al*., 2008). Moreover, the unfolded protein response of the endoplasmic reticulum (UPR^ER^) and the mitochondria (UPR^mito^) is also influenced by neuronal function (Durieux *et al*., 2011; Sun *et al*., 2011; Singh & Aballay, 2012; Taylor & Dillin, 2013).

Perhaps the most well‐studied environmental factor that influences aging is nutrient availability. Dietary restriction is a robust means of initiating a system‐wide response that improves the efficiency of various homeostatic cellular metrics, ameliorating stress and ultimately resulting in lifespan extension. The DR response is highly conserved across organisms. The effects of DR on lifespan have been known since the 1930s and for years people have sought chemical compounds that could induce DR mechanisms (Ingram *et al*., 2006). The potential discovery of DR mimetics has been compelling since the publication of the first such candidate highlighted the potential for obtaining the advantages associated with DR, namely improved physiology, without having to adopt a restrictive and dissatisfying diet (Lane *et al*., 1998).

Dietary restriction can be elicited by altering nutrient signals. For example, chemical structures that mimic dietary components, endogenous metabolites, and perturbation of specific neurotransmitter pathways have all been shown to shift organismal physiology into a DR state (Petrascheck *et al*., 2007; Williams *et al*., 2009; Lucanic *et al*., 2011; Chin *et al*., 2014). Dietary restriction is also influenced by sensory perception, and primary sensory neurons have been shown to be integral to achieving the DR response to low nutrients (Bishop & Guarente, 2007).

*Caenorhabditis elegans* has proved to be a good model organism for the discovery of chemical compounds and plant extracts that modulate aging (Collins *et al*., 2006; Lucanic *et al*., 2013a; Carretero *et al*., 2015). In an effort to identify novel modulators of DR, we have undertaken small‐molecule screens in *C. elegans* to identify new chemical structures that can delay aging. Here, we describe one such novel chemical, NP1, and outline its mechanism of action. NP1 interacts with a sensory pathway that informs on nutrient availability in *C. elegans*. We find that NP1 promotes glutamatergic signaling through a glutamate‐gated chloride channel and that this mimics the loss of a muscarinic acetylcholine signal which normally functions to inhibit this pathway when food is abundant. These results demonstrate that chemical modulation of nutrient sensory pathways can illicit organism‐wide physiological responses associated with dietary restriction even in the presence of a normal diet.

## Results

### A small‐molecule screen for longevity compounds identified a new biologically active chemical structure

**Figure 1:**
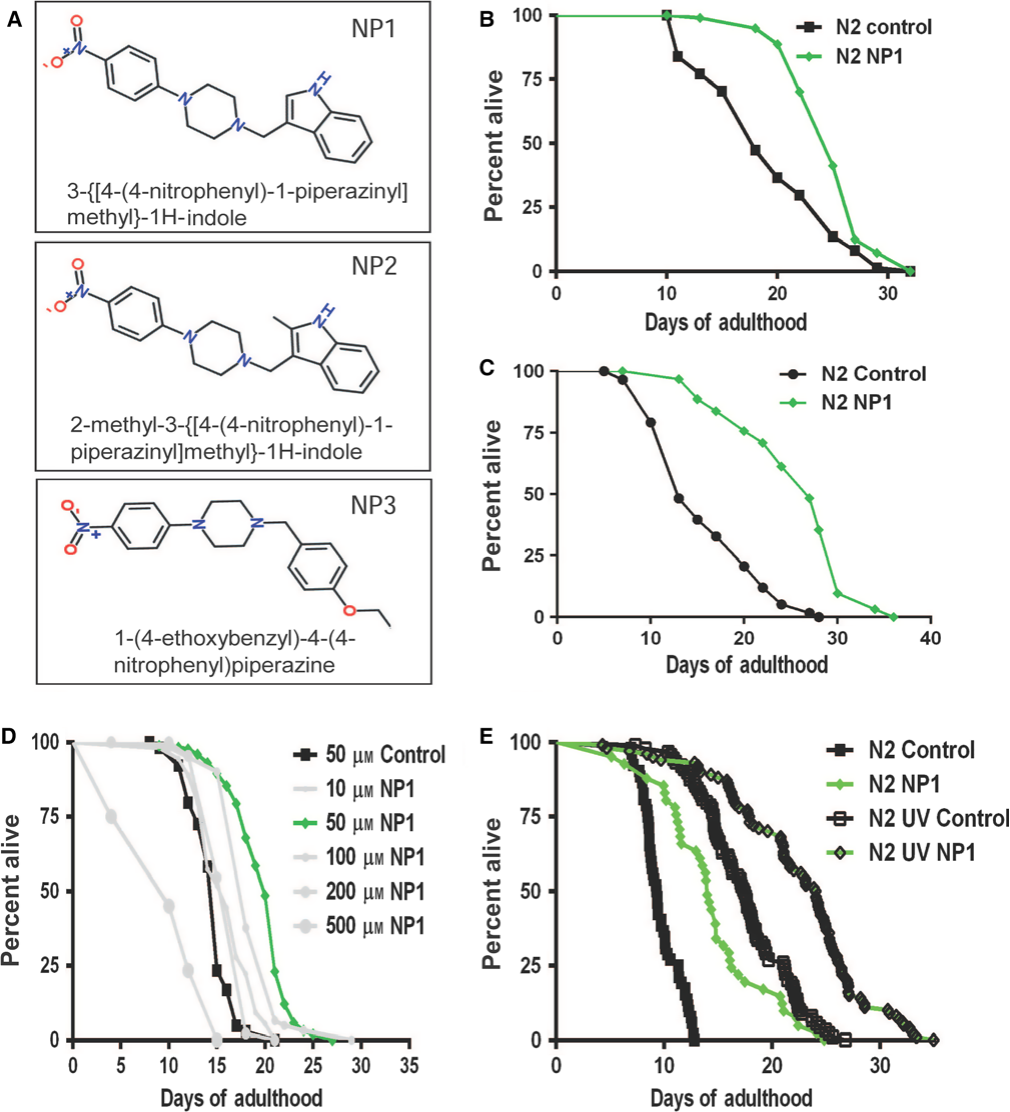
NP1 promotes longevity. (A) Three chemicals, all containing a nitrophenyl piperazine motif, were identified as promoting lifespan in a small‐molecule screen performed in *C. elegans*. Treatment of wild‐type animals with NP1 from hatching (B) or from the young adult stage (C) had a significant positive effect on lifespan (*P* < 0.0001 compared to control for (B) and *P* < 0.0001 compared to control for (C). (D) Dose response of wild‐type *C. elegans* to NP1 identified 50 μm as the peak dosage for promoting lifespan (*P* < 0.0001 compared to control treatments for 10, 50 and 100 μm NP1). (E) Survivorship graph of NP1 effect on live or UV‐irradiated bacteria (data represent pooled data from four replicates of non‐UV‐treated and eight replicates of UV‐treated bacteria. *P* < 0.0001 for N2 UV NP1 compared with N2 UV Control). See also Table S1.

To identify novel, biologically active chemicals that modulate aging‐related phenotypes, we screened 30 000 synthetic, structurally diverse drug‐like compounds for lifespan extension in the free‐living nematode *C. elegans* (Fig. S1). We identified over five hundred primary hits, of which one hundred and eighty were selected for re‐testing. Re‐testing of the compounds was performed in triplicate and was re‐arrayed in 96‐well assay plates to help mitigate positional plate effects. Binning of the re‐test results indicated that the primary hits from the chemical screen outperformed control wells (Fig. S1). Top hits from the re‐test assay were tested under standard *C. elegans* culture conditions (single plate). While most of the 57 identified chemicals that reproducibly induced lifespan extension were structurally diverse, three were closely related. These compounds all contained a nitrophenyl piperazine backbone (Fig. 1A), and they were therefore named **n**itrophenyl **p**iperazine‐containing compounds 1–3 (NP1‐3). NP1 was determined to be the most potent of these compounds inducing a robust lifespan extension regardless of whether exposure was initiated just prior to hatching (Fig. 1B) or at the young adult stage (Fig. 1C). NP1 exhibited a dose–response effect on lifespan that peaked at 50 μm (Fig. 1D).

### NP1 acted through a DR‐type mechanism

*Caenorhabditis elegans* are bacterivores and are generally cultured on living bacteria. Recent research in *C. elegans* has highlighted the impact of the metabolism of the bacterial food as contributing to the effect of chemical treatments intended only for the worms (Onken & Driscoll, 2010; Cabreiro *et al*., 2013). To determine whether the metabolic activity of the bacterial food was at all causative to the NP1 effect, we tested NP1 on worms cultured on bacteria pretreated with ultraviolet radiation (UV) (Fig. 1E). Under these conditions, NP1 was still effective at prolonging lifespan relative to untreated animals, demonstrating that NP1 effect did not require metabolically active bacteria.

**Figure 2:**
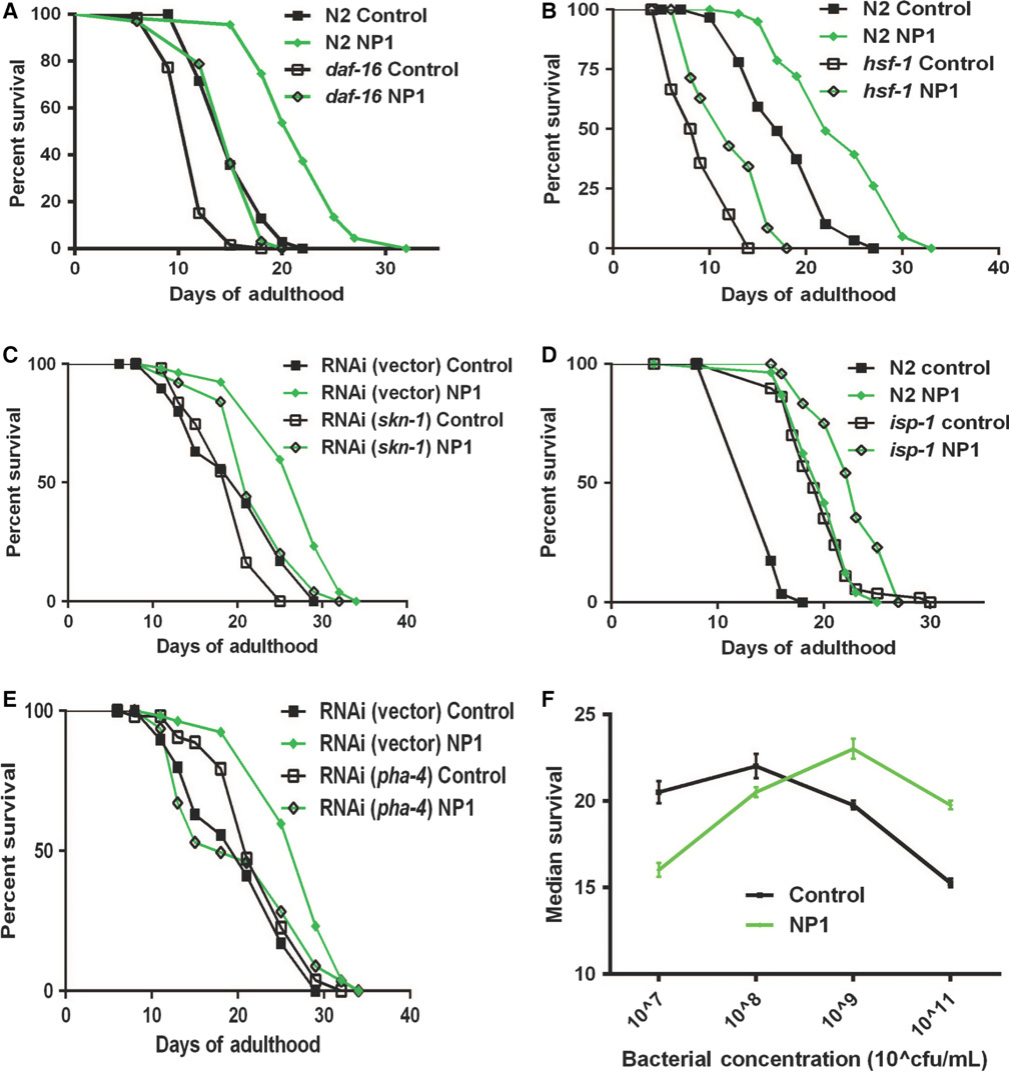
NP1 extends lifespan through a DR‐related mechanism. (A–E) Representative survivorship plots demonstrating the interaction of NP1 with known lifespan‐promoting pathways. NP1 extended the lifespan of *daf‐16* (*P* < 0.0001 compared to *daf‐16* control‐treated) (A) and *hsf‐1* (*P* < 0.0001 compared to *hsf‐1* control‐treated) mutants (B) and also of animals undergoing RNAi of *skn‐1* (*P* = 0.0005 compared to *RNAi(skn‐1)* control treated) (C). *isp‐1* long‐lived mutants also responded to NP1 (D) with further lifespan extension (*P* < 0.0001 compared to *isp‐1* control treated). Animals fed dsRNA of the FOXA‐type transcription factor PHA‐4 did not respond to NP1 with lifespan extension (*P* = 0.7905 compared to *RNAi(pha‐4)* control treated) (E). Graph showing the relationship between food concentration and the median survival of TJ1060 worms under control‐ and NP1‐treated conditions (average of four independent replicates) (F). See also Table S1.

To begin to determine the mechanism of the lifespan extension induced by NP1, we first tested whether it acted through the pathways known to influence lifespan in *C. elegans*. DAF‐16, the transcription factor effector of insulin‐/IGF‐like signaling, was not required for the effect of NP1, as null mutants still responded to NP1 with lifespan extension relative to control‐treated animals (Fig. 2A). Mutants or RNAi of other major stress‐response transcription factors (HSF‐1 and SKN‐1) that are known to mediate lifespan extension also showed lifespan extension from the addition of NP1 compared to nontreated controls. *hsf‐1* mutants and *hsf‐1* RNAi‐treated animals both responded well to NP1, while *skn‐1* RNAi‐treated animals responded with a significant but attenuated lifespan extension compared to the wild‐type response (Fig. 2B,C). Interpretation of these results is less clear as these genes are essential for viability, and thus, only hypomorphic alleles and/or RNAi treatments were assayed. RNAi results additionally require careful interpretation because some tissues, including neurons, are known to respond poorly, leading to possible tissue‐specific effects resulting from mosaic or incomplete knockdown of the target. Because of these caveats, our results cannot completely exclude these two genes from being involved in the response to NP1. NP1 also further extended the lifespan of a long‐lived mitochondrial mutant (*isp‐1)* (Fig. 2D). However, NP1 failed to extend the lifespan of worms fed dsRNA of the FOXA‐type transcription factor *pha‐4* (Fig. 4E). Because PHA‐4 was previously identified as being required for the lifespan‐extending effects of DR, this suggested that NP1 may elicit DR (Panowski *et al*., 2007). To further test this, we conducted assays with NP1 in a dietary restriction protocol that directly limits the bacterial food available. We observed a lifespan response to food concentrations on a level with previously reported values (Chen *et al*., 2009). NP1 was effective at extending lifespan in conditions of food abundance, but not under strong DR conditions (Fig. 2F). The dependence on high food concentrations for NP1 to induce lifespan extension indicates that NP1 acted by promoting a DR response.

### NP1 decreased the pharyngeal muscle contraction frequency through glutamatergic signaling

There are many ways of eliciting the DR response, from directly reducing food intake to activating the downstream pathways that cause the physiological changes that promote extended lifespan. We first tested whether NP1 influenced feeding (and therefore food intake) by measuring the pharyngeal muscle pumping rates. *Caenorhabditis elegans* are bacterivores that feed by pumping their pharyngeal muscles, which draw food into the pharyngeal lumen. This food is then ground up and passed on to the intestine (Avery & Horvitz, 1989). NP1‐treated animals showed a subtle but significant reduction in pumping rate relative to control‐treated animals (Fig. S2). Maximum effect was observed after 3 h and was similar in acute or chronically treated animals (Fig. S2). We also tested the effect of the chemical in an *in vitro* assay with dissected heads and found a similar inhibitory effect on pumping which started at lower concentrations. Because the dissected head assay removes the cuticle, a likely barrier to chemical entry, this result suggests that the *in vivo* concentration of the compound in our standard 50 μm assays is likely to be in the nanomolar to low micromolar range (Fig. S2).

**Figure 3:**
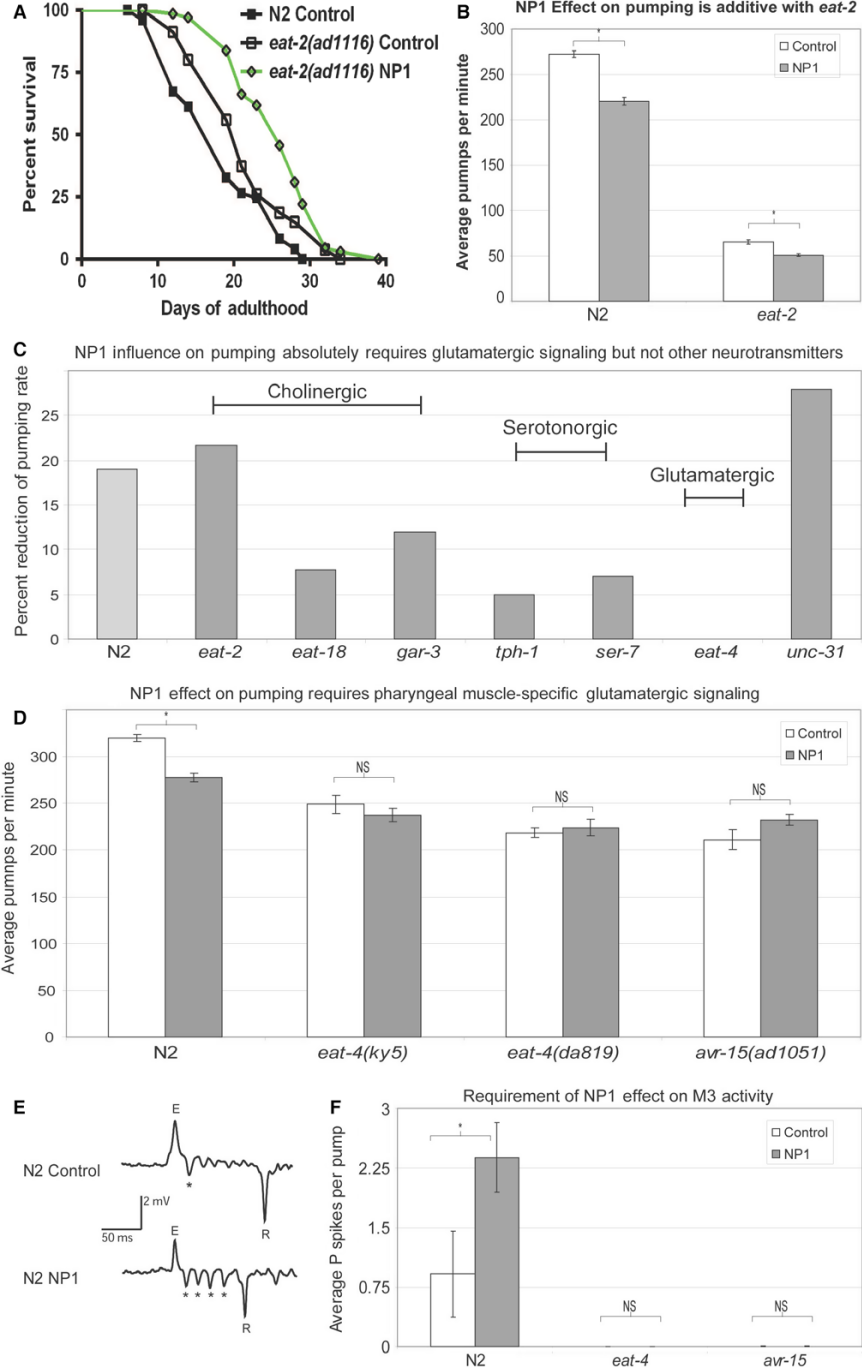
NP1 effect on feeding behavior acts in parallel with *eat‐2* but requires glutamatergic signals to the pharyngeal muscles. (A) Survivorship graph showing the lifespan‐extending effect of NP1 on *eat‐2* (*P* = 0.0018 compared to *eat‐2* control treated). A graphical representation of pumping rates is presented in (B) that demonstrates the slight but significant decrease in pumping observed from NP1 treatment on wild‐type was also observed in NP1‐treated *eat‐2* animals. (C) Graphical representation of the effect (100‐(NP1 treated/control treated)*100) of NP1 on mutants with defects in neurotransmission to the pharyngeal muscle. The mutants are grouped by the type of neurotransmitter effected. (D) Graph of the pumping rates of glutamatergic mutants treated with control or NP1. (E) Representative filtered electropharyngeogram (EPG) traces recorded using the NeuroChip with live whole *C. elegans* either control or NP1 treated. Excitation [E], relaxation [R], and P spikes [*] are annotated. (F) Graphical representation of the average numbers of P spike events from 60 s traces of wild‐type and glutamatergic mutants treated with control (DMSO) or NP1. EPG traces were annotated with AutoEPG software. Animals assayed; N2: four control and six NP1 treated, *eat‐4*: six control and three NP1 treated, avr‐15: eight control and five NP1 treated. Pumping rate data shown were collected by video capture with the subsequent video playback at 0.25 speed and the manual counting of pharyngeal bulb contractions, except for the data in (C) which were collected by counting pumps in real time. Error bars show the standard error of the mean. For the averaged number of P spikes, a two‐way ANOVA was used to test the significance between the averaged values, for N2 (*P* < 0.01).

Despite the decrease in average feeding rates, we were unable to detect significant differences in food intake between NP1‐treated and control worms (Fig. S2). As NP1 did not appear to decrease feeding rate *in vivo* and did not cause worms to appear starved or poorly fed, we thought it unlikely that the NP1 pumping rate defect could cause such a profound increase in lifespan. Genetic mutations that result in DR from mechanical impairments of feeding are well known in *C. elegans* (Lakowski & Hekimi, 1998). EAT‐2 is an acetylcholine receptor subunit responsible for promoting the rapid pumping of pharyngeal muscle (Raizen *et al*., 1995; McKay *et al*., 2004). *eat‐2* mutants exhibited profoundly decreased pumping rates, appeared starved, and were long‐lived relative to wild‐type (Fig. 3A). We found that, despite the much greater effect of the *eat‐2* mutation on pumping rates, the mutation did not confer a greater magnitude of lifespan extension than NP1 treatment. Furthermore, treating the *eat‐2*‐mutant worms with NP1 resulted in an additive lifespan extension; the effects on pumping were also additive (Fig. 3A,B). Collectively, these results indicated that the longevity effect of NP1 did not simply result from limiting nutrient intake through a decreased feeding rate.

Pharyngeal pumping is a well‐studied neuromuscular activity that consists of the isolated pharyngeal‐specific nervous system controlling the pharyngeal muscle network which is collectively linked through gap junctions (Franks *et al*., 2006; Avery & You, 2012). The system utilizes several distinct neurotransmitter pathways. To determine whether NP1 was acting through the modulation of a specific neurotransmitter signaling pathway, we tested a panel of neurotransmission mutants. We found that mutants completely devoid of glutamatergic signaling, through the loss of the glutamate transporter (EAT‐4) required for loading glutamate into synaptic vesicles (Lee *et al*., 1999), failed to respond to the chemical (Figs 3C,D and S2), while mutants defective in other implicated pathways, such as cholinergic, serotonergic, and neuropeptide signaling, all responded to NP1 with a reduction in pumping rate (Fig. 3C). We found that similar to glutamatergic null mutants, the pharyngeal muscle‐specific glutamate receptor mutant *avr‐15* (Dent *et al*., 1997) also failed to respond to NP1 (Fig. 3D). These results demonstrate that the decreased pumping rate effect of NP1 requires glutamatergic neurotransmission to the pharyngeal muscle.

### NP1 promotes glutamatergic signaling through the glutamate‐gated chloride channel AVR‐15

The major function of glutamatergic signaling in the pharyngeal nervous system is through the M3 neurons that drive the inhibitory postsynaptic potentials (IPSPs) that facilitate re‐polarization of the pharyngeal muscle (Avery, 1993; Raizen & Avery, 1994). These IPSPs (P spikes) can be visualized in the electrophysiological traces of neuromuscular function, which are known as electropharyngeograms (EPGs) (Fig. 3E). The glutamate‐gated chloride channel AVR‐15 is expressed in the pharyngeal muscle and is absolutely required for these IPSPs (Dent *et al*., 1997). To test whether NP1 activates or inhibits signaling through AVR‐15, we utilized the NeuroChip microfluidic device (Hu *et al*., 2013) to record EPGs and examine the effects of NP1 on these IPSPs. If NP1 inhibits glutamate signaling through AVR‐15, then we would predict that NP1 would suppress P spikes; if NP1 promotes signaling, we would expect to observe more P spikes. Significantly more P spikes per action potential were recorded in NP1‐treated animals than in the control‐treated animals (Fig. 3F). Consistent with previous observations, we did not detect P spike‐type IPSPs in either *eat‐4* or *avr‐15* mutants. Additionally, we did not detect a significant difference in the number of P spikes per action potential in NP1‐treated *eat‐4* or *avr‐15* mutants relative to the control‐treated animals. Collectively, these data indicate that NP1 promotes signaling through AVR‐15 in the pharyngeal muscle.

### NP1‐induced longevity also requires glutamatergic signals to the pharyngeal muscles

**Figure 4:**
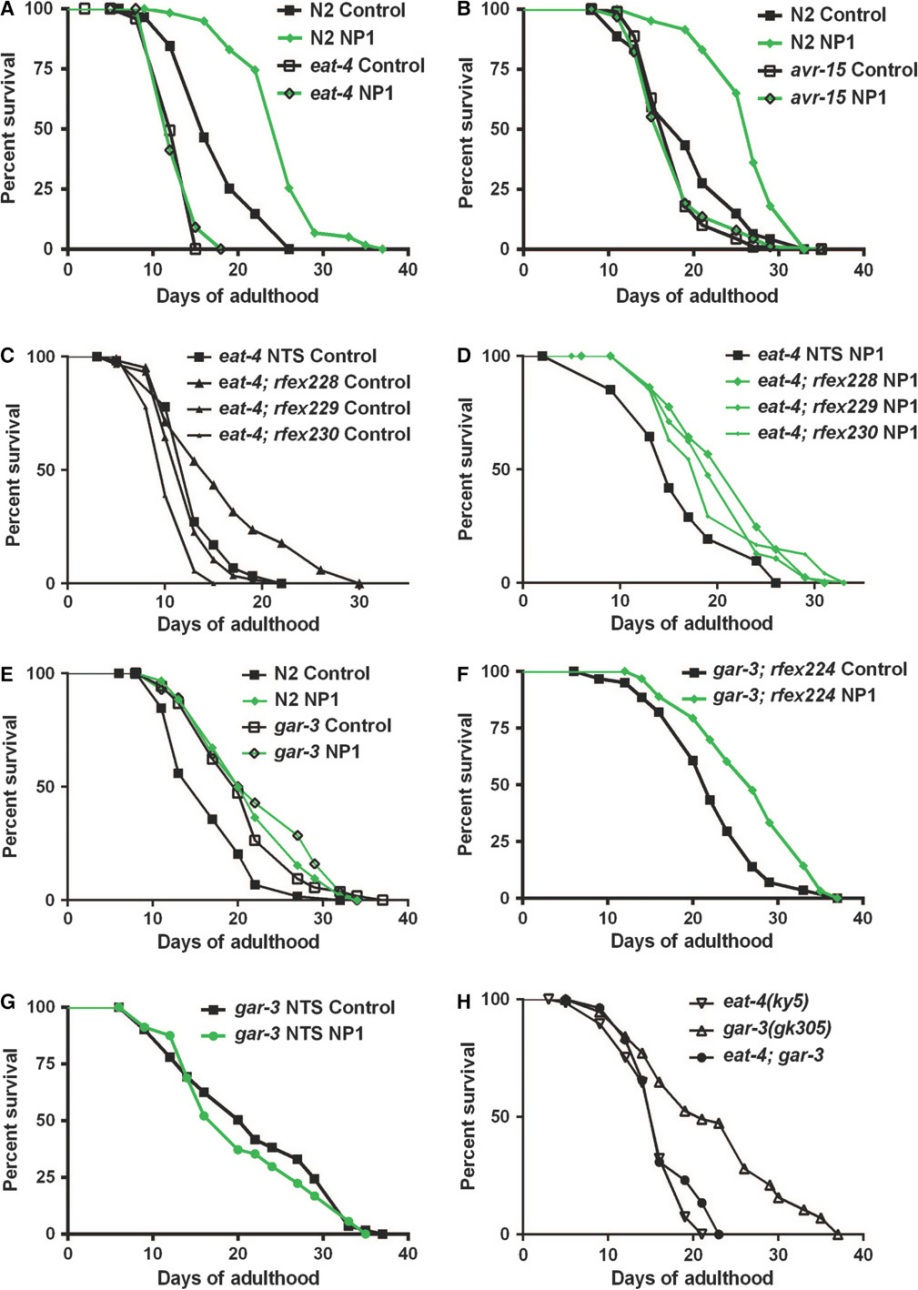
The NP1 effect on lifespan requires glutamatergic signaling downstream of a metabotropic‐type acetylcholine receptor. (A, B) Representative survivorship graphs demonstrating that *eat‐4* (*P* < 0.0001 short‐lived) (A) and *avr‐15* (*P* = 0.0448 short‐lived) (B) mutants did not exhibit extended lifespans relative to wild‐type, nor do they respond to NP1 with lifespan extension (*P* = 0.7959 and 0.8721, respectively, compared to the untreated controls). (C, D) Survivorship graphs of *eat‐4(ky5)* mutants expressing *eat‐4* in the NSM neurons (*rfex228‐230[Ptph‐1::eat‐4]*) and their nontransgenic siblings (NTS). The lifespans of these worms were assayed under both control‐ (C) [compared to NTS controls: *P* = 0.0152 (*rfEx228*), *P* = 0.197 (*rfEx229*), and *P* = 0.0478 (*rfEx230*)] and NP1‐treated (D) conditions [compared to NP1‐treated NTS:* P* = 0.0049 (*rfEx228*), *P* < 0.0001 (*rfEx229*), and *P* < 0.0001 (*rfEx230*)]. (E–H) Survivorship graphs indicated the involvement of GAR‐3 in the NP1‐mediated signaling pathway. The putative null mutants *gar‐3(gk305)* were long‐lived relative to wild‐type (*P* < 0.0002 compared to wild‐type) and did not respond to NP1 with further lifespan extension (*P* = 0.1497 compared to *gar‐3* control) (E). Tissue‐specific expression of *gar‐3* in the pharyngeal muscle (*rfex224[Pmyo‐2::gar‐3]*) was sufficient to rescue the NP1 response (*P* = 0.0002 compared to *gar‐3; rfEx224* control) (F), while the NTS did not respond to NP1 with lifespan extension (*P* = 0.5596 compared to *gar‐3* NTS control treated) (G). *eat‐4* was epistatic to *gar‐3* as the *eat‐4; gar‐3* double mutant had the phenotype of the *eat‐4* single mutant (*P* = 0.1365 compared to *eat‐4* control) (H). See also Table S1.

We next tested whether the longevity effect of NP1 also required functional glutamatergic signaling. Indeed, both glutamatergic null animals (*eat‐4*) and the pharyngeal muscle glutamate receptor mutants (*avr‐15*) failed to respond to NP1 with lifespan extension (Fig. 4A,B). This also demonstrated that the loss of glutamatergic signaling did not extend lifespan relative to wild‐type controls (Fig. 4A,B). This was surprising because these glutamatergic mutants exhibited a stronger pumping rate decrease than the NP1‐treated animals (Fig. 3D). Collectively, these results show that the relatively minor reduction in pumping rate associated with a loss of glutamatergic signaling is not sufficient to extend lifespan. This finding supports our previous observations that the decrease in pumping rate observed in NP1‐treated animals is unlikely to be causative for the lifespan extension. However, an alternative explanation for the lack of extended lifespan in glutamatergic mutants, whether or not NP1 is present, could be that glutamatergic mutants are generally defective in their response to DR. To test this possibility, we examined the lifespan of *eat‐4‐* and *avr‐15*‐mutant populations cultured with variable food concentrations. This was not the case, because both mutant populations showed a robust lifespan extension response to DR (Fig. S3). Taken together, our results indicate that glutamatergic signals are not absolutely required for DR, yet can be utilized to promote DR, supporting the existence of redundant DR activation pathways.

The results from our lifespan analysis indicate that NP1 requires glutamatergic signaling to the pharyngeal muscle but does not simply act through inhibiting these signals. This is complementary to the electrophysiological analysis of NP1‐treated worms, which suggested that NP1 acted through promoting glutamatergic signaling to the pharyngeal muscle. To further test this hypothesis, we utilized an *eat‐4*‐mutant background and ectopically expressed EAT‐4 in a pair of nonglutamatergic motor neurons that synapse onto the pharyngeal muscle (the NSMs). Expression of EAT‐4 in a neuron is the defining feature of a glutamatergic neuron (Serrano‐Saiz *et al*., 2013) and is therefore expected to be sufficient to cause the loading of glutamate into endogenous synaptic vesicles for release onto the pharyngeal muscle. Furthermore, NSM neurons are not required for the lifespan‐extending effect of NP1 (Fig. S3). We found that expression of EAT‐4 in the NSMs had a relatively weak and variable effect on lifespan but that it resulted in rescued sensitivity to the lifespan‐extending effects of the chemical NP1, which is lost in *eat‐4* mutants (Fig. 4A,C,D). This result again indicated that NP1 acted to increase lifespan through an increased glutamatergic signaling to the pharyngeal muscle, regardless of the neural origin of the glutamate signal, and further suggested that NP1 likely acts downstream of the M3 neurons.

### Mutations in the M1/M3/M5‐type muscarinic acetylcholine receptor *gar‐3* mimic the lifespan effects, but not the pumping effect of NP1

NP1 activates a DR effect on lifespan by increasing glutamatergic signaling to the pharyngeal muscle. This suggested that NP1 may impinge on a sensory pathway involved in nutrient detection, perhaps by mimicking a signaling state that occurs in situations of low food abundance. The metabotropic‐type acetylcholine receptor GAR‐3 has been reported to have dual roles in the pharyngeal muscle contraction (Steger & Avery, 2004) and has also been shown to be involved in starvation signaling (You *et al*., 2006). We found that *gar‐3* mutants [including the nonsense allele *gar‐3(gk305)*] were long‐lived relative to wild‐type and they failed to respond to NP1 with a further lifespan extension (Figs 4E and S3). This result is consistent with NP1 acting by inhibiting or phenocopying an inhibited GAR‐3. Interestingly, we found that *gar‐3* mutants responded to NP1 with decreased pumping, demonstrating that the lifespan and pumping phenotypes of NP1 can be dissociated (Fig. S3).

Recently, several reports have described the important roles for GAR‐3 in neurons, mediating multiple roles in acetylcholine signaling, including acting as an extrasynaptic acetylcholine detector in ventral motor neurons (Hendricks *et al*., 2012; Chan *et al*., 2013). GAR‐3 is expressed in neurons and the pharyngeal muscle, with particularly high expression in the terminal bulb of the pharynx. To test whether GAR‐3 was acting in neuronal or muscle tissue, we expressed GAR‐3 in the pharyngeal muscle and examined the response to NP1. The pharyngeal muscle expression of GAR‐3 was sufficient to restore the response to NP1 (Fig. 4F,G).

**Figure 5:**
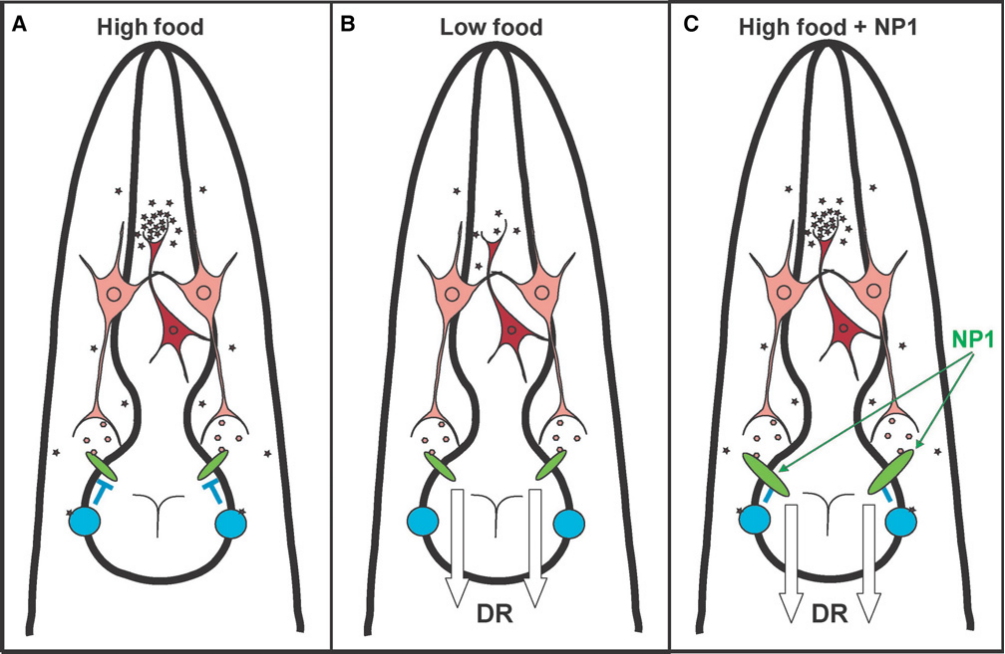
Model of the nutrient detection signaling pathway antagonized by NP1. (A–C) A model of the different pharyngeal muscle signaling states described. (A) When food is abundant, pharyngeal cholinergic neurons (shown in red) are highly active and release acetylcholine. The high levels of acetylcholine result in activation of the metabotropic acetylcholine receptor GAR‐3. GAR‐3 inhibits signaling through the glutamate‐gated chloride channel, and this signaling suppresses DR. (B) When food is scarce, acetylcholine levels are low and do not activate GAR‐3. High signaling through AVR‐15 then elicits a DR response. (C) NP1 addition promotes signaling through AVR‐15 and this elicits DR even in the presence of abundant food by masking or inhibiting the effect of GAR‐3. Green ovals represent AVR‐15, and blue circles represent GAR‐3. Pink neurons represent the M3 neurons, while pink hexagons represent glutamate. The red neuron represents cholinergic neurons (a single neuron is shown for clarity), while red stars represent acetylcholine.

As we had identified two signaling pathways with different lifespan phenotypes (*eat‐4* and *gar‐3*), which were linked by their failure to respond to NP1 with lifespan extension, we next examined the order of these mutants by analyzing the lifespan phenotype of the double mutants. We found that the *eat‐4;gar‐3* double mutants had the short lifespan phenotype of the *eat‐4* single mutants and not the long lifespan phenotype of the *gar‐3* single mutants (Fig. 4H). This demonstrates that *eat‐4* is epistatic to *gar‐3* and therefore acts further downstream in the linked pathway. Collectively, our results demonstrate that NP1 extends lifespan in a DR‐like manner by promoting glutamatergic signals to the pharyngeal muscle. In the absence of NP1, this response is likely regulated through acetylcholine signaling by the metabotropic‐type acetylcholine receptor GAR‐3 (Fig. 5).

## Discussion

The detection of nutrients through primary sensory neurons is an important component of dietary restriction in metazoans, and it may represent an attractive target for pharmaceuticals in eliciting DR in animals on a normal diet. Sensory perception pathways allow for the rapid response of an organism to an external stimulus. These systems may lead to more rapid responses to changing food environments than secondary pathways such as cellular detection of energy levels or the detection of absorbed dietary components and their levels. Primary sensory systems have the advantage pharmacologically of being more specific targets than intracellular energy detection pathways such as mTOR or AMPK. External or primary sensory systems seem to be geared toward specific stimuli, while intracellular components tend toward being utilized as nodes that respond to multiple stimulus and cellular conditions.

We suggest that the GAR‐3/AVR‐15 pathway described here is part of a primary sensory system that detects food availability in *C. elegans*. We found that NP1 acts through DR to extend lifespan (Fig. 2F) and that a profile of the DR response is shifted toward higher food concentrations in the presence of NP1, suggesting an attenuated food perception. GAR‐3 loss‐of‐function mutants also show extended lifespan and are not further extended by NP1 (Figs 4E and S3). This indicates that GAR‐3 signaling negatively regulates the DR pathway promoted by NP1. GAR‐3, which we link to DR and has previously been shown to be important in responding to nutrient conditions (You *et al*., 2006), has also been shown to act as an extrasynaptic detector of acetylcholine levels (Chan *et al*., 2013). Our model to fit these observations suggests that food abundance induces an increased acetylcholine, which is detected by GAR‐3 on the pharyngeal muscles (Fig. 5). This is consistent with the fact that acetylcholine is the primary excitatory neurotransmitter that influences the pace of feeding.

This model of acetylcholine monitoring at the pharyngeal muscle explains the additive effect on lifespan observed between the *eat‐2* mutation and NP1 (Fig. 3A). *eat‐2* mutants are not expected to have decreased acetylcholine levels and are only defective for the response to acetylcholine. This model, however, does not explain the significance of the interaction between GAR‐3 and AVR‐15. Why should a muscarinic‐type receptor require a chloride channel to have a physiological effect? This is still not clear. Previous work has highlighted a complex role for GAR‐3 in regulating multiple activities of the pharyngeal muscle, including calcium ion dynamics and membrane re‐polarization timing in pharyngeal muscle (Steger & Avery, 2004). However, muscarinic‐type receptors are well known to control ion channel function in vertebrate cells (Pfaffinger *et al*., 1985; Shi *et al*., 2004), including heart muscle that shares remarkable similarities with nematode pharyngeal muscle (Mango, 2007). Our lifespan data demonstrate that the loss of GAR‐3 muscarinic receptor signaling and the chemical NP1 act through the ion channel AVR‐15. However, the effect on pumping from these two different manipulations are distinct, because NP1 reduces pumping significantly while we could not detect a significant difference in pumping rate between wild‐type and *gar‐3* mutants (Fig. S3). This indicates that while the effect of removing *gar‐3* results in a relatively subtle increase in the activity of AVR‐15, NP1 administration has a much more profound effect on the channel. Thus, a subtle increase in AVR‐15 function is sufficient to induce the DR response, while a strong AVR‐15 increase in activity causes an additional effect on pumping rate.

The specificity of the NP1 effect on lifespan as requiring *avr‐15* and mimicking *gar‐3* mutants suggests that NP1 acts through specific interaction with a single molecule or molecular complex and rather than having multiple targets. NP1‐3 all contain a piperazine domain (Fig. 1), and piperazine itself has long been known to be a weak agonist of GABA‐type chloride channels, which themselves are related to glutamate‐gated chloride ion channels. We have tested the effects of piperazine on pharyngeal pumping and lifespan and did not observe the effects that were similar to NP1 (data not shown). High doses of piperazine did inhibit pumping, but general paralysis was the most apparent phenotype with piperazine at millimolar concentrations. Paralysis was not observed from NP1 treatment, and additive experiments seemed to indicate that they do not enhance each other's effects, suggesting distinct mechanisms. There are a large number of other pharmacologically active piperazine‐containing molecules, some of which are involved in promoting or inhibiting monoamine signaling, as well as other biological pathways. Further analysis is needed to determine the exact molecular target of NP1.

Chemical screens can uncover the modulators of aging not identified in genetic screens. Here, we show that a screening of diverse, synthetic compounds for lifespan extension reveals a novel nutrient sensing pathway. While the specificity of the sensory system targeted by NP1 suggests that this chemical is perhaps unlikely to alter nutrient detection in mammals, recent work has shown that sensory systems are at play in regulating mammalian longevity and that they are not restricted to nutrient perception (Riera *et al*., 2014). Our work further demonstrates that there are likely to be many potential targets for the development of drug‐like mimetics of diet restriction.

## Materials and methods

### *Caenorhabditis elegans* culture conditions and strains

Nematodes were maintained as previously described (Brenner, 1974). The following previously described strains and alleles were used in this study: N2*; gar‐3(gk305); gar‐3(vu78); daf‐16(mu86); hsf‐1(sy441); isp‐1(qm150); osm‐3(p802); eat‐18(ad1110); tph‐1(mg280); ser‐7(tm1325); unc‐31(e928); eat‐2(ad1116); eat‐4(ky5); eat‐4(ad819); avr‐15(ad1051);* TJ1060*[spe‐9 (hc88);fer‐15(b26)]; zdIs13[Ptph‐1::GFP]*.

### Generation of transgenic *Caenorhabditis elegans* lines

To obtain worm lines that express EAT‐4 in the NSM motor neurons, we used polymerase chain reaction (PCR) to isolate the promoter region of *tph‐1* (primers f1: ccacttgagcttttccactgatcaacc and r1: atgattgaagagagcaatgctacctaaaaaccaaag) and the *eat‐4* coding region (primers f1: CAAGCCTCGTTCCATGACGACAT and r1: ctggtagaaaagcagaggagaagaaggc); these DNA fragments were then fused by PCR (any overlap region of primers is omitted for clarity) and injected into the gonads of young adult hermaphrodites (Hobert, 2002). Individual progeny of these injected animals were used to find transgenic lines, three of which were selected for further study (*rfEx228‐230*) based on the expression of a co‐injection marker.

To obtain the pharyngeal‐specific expression of GAR‐3, the above strategy was again employed using the promoter from *myo‐2* (primers f1: ggtggtggacagtaactgtctgt and r1: ttctgtgtctgacgatcgagg) and the *gar‐3* genomic region (primer f1: cagtcctcttcgttggggaatgctg and r1: gacatgggcgacttcttaatacagatgttctcaaaca). Again using microinjection, two transgenic lines were obtained for further study *(rfEx224‐225)*.

### Lifespan assays

Regular lifespan assays (non‐multiwell, high‐throughput, or automated lifespan machine based) were performed as previously described (Lucanic *et al*., 2013b). In brief, synchronized (from 2‐h egg lays) worm populations were added to 3 cM NGM plates supplemented with 10 μg/mL 5‐fluoro‐2′‐deoxyuridine (FUdR) at day one of adulthood. Animals were scored and transferred to fresh plates every other day through the first two weeks, with weekly transfers after the first two weeks. Lifespan assays were performed at 20 °C. Feeding RNAi lifespans were performed as described above except that worms were shifted to and then maintained on RNAi plates (NGM supplemented with 100 μg/mL + 400 μm IPTG) containing bacteria expressing the specific RNAi vector or the control vector (L4440) on day one of adulthood.

For multiwell assays (re‐test assay), TJ1060 animals were used (no FUdR) and incubated at 25 °C from hatching. Three of the control plates were scored at days 14, 16, and 18 of adulthood. At day 18, we discovered approximately 95% mortality on the scored control plates. All other plates (control and test) were then completely scored for mortality.

Automated lifespan machine assays were performed essentially as previously described (Stroustrup *et al*., 2013) except for the timing of FUdR addition, amount of FUdR, and the point at which they were loaded onto the scanners. Specifically, worms were loaded onto the scanners for lifespan analysis at day four of adulthood after initiating FUdR treatment (10 μg/mL) at day one of adulthood and transferred to fresh FUdR‐containing plates on day two and day four of adulthood. These modifications were made in order to delay FUdR treatment until after somatic development was complete, to minimize FUdR levels in the experiment, and to avoid moving larva to the scanner plates. For all lifespan analyses, comparisons of survival were made with GraphPad Prism^™^ software, and *P* values were generated by the log‐rank Whitney test of significance.

### Chemical screen for enhanced lifespan

For the high‐throughput screen cultures, ten mass culture plates (10 cM NGM with concentrated OP50) with approximately 25 000 gravid TJ1060 animals were collected in S basal. These plates were treated with a hypochlorite solution and then washed three times and incubated at low density in S basal for 16 h in glass dishes. Resulting L1 arrested worms were resuspended at one worm/microliter in S basal. The worms were then dispensed into 96‐well plates containing NGM and concentrated OP50 at ten worms per well with a Multidrop 384 reagent dispenser (Thermo Scientific). The plates were then incubated for 48 h at 25 °C to reach adulthood. Chemicals were then dispensed onto each well to a final concentration of 50 μm with a Biomek FX Liquid Handler (Beckman Coulter). The plates were then dried in a sterile hood and then incubated at 25 °C until control plates exhibited 95–100% mortality (18–21 days). The plates were scored manually with a stereomicroscope.

### Chemical treatment of *Caenorhabditis elegans*

For standard assays, chemicals were added to food‐containing (OP50 bacteria) 3 cm culture plates (standard NGM agar) at least one day prior to use to allow for distribution of the chemical throughout the culture plate. DMSO concentrations in NP1 assays were kept at 0.25%, except for screening assays that contained DMSO at 0.53%. DMSO levels were always matched in control experiments. Unless otherwise stated, chemical treatment was initiated on 1st day of adulthood and maintained throughout the entire lifespan. NP1‐treated worms appeared healthy and well fed with no obvious abnormalities other than a slight (6‐ to 12‐h) developmental delay observed when worms were hatched in the presence of the chemical (data not shown).

### Pharyngeal pumping rate measurements

Unless otherwise stated, feeding behavior in chemical‐treated animals was assayed in second‐day adults, after 24 h of chemical treatment starting on the first day of adulthood. For chemical treatments, the worms were incubated with chemical and then moved onto a standard food‐containing culture plate for five minutes prior to measurements. Pumping rates were either counted in real time over 30 s or were first captured in 10‐s videos and then later scored by counting the pharyngeal contractions in playback at one‐quarter speed. The specific method used in each experiment is indicated in the figure legend. At least 20 animals were tested for each genotype and condition. Student's *t*‐test was used to determine the *P* values.

### Electrophysiology

Electropharyngeogram (EPG) recordings were carried out with whole animals in the NeuroChip microfluidic device as previously described (Hu *et al*., 2013). Briefly, the worms were placed in the device in Dent's solution supplemented with 5 mm 5‐HT (to stimulate pumping) and recorded for 2–5 min. Recorded EPG traces were analyzed using the AutoEPG software (Dillon *et al*., 2009), which annotates and compiles individual features of the traces such as P spikes. Two‐way ANOVA within GraphPad Prism^™^ software was used to test significance.

### Laser ablation

Laser ablation was performed essentially as previously described (Fang‐Yen *et al*., 2012). Briefly, arrested first larval‐stage animals were mounted on microscope slides with 2% agarose pads and anesthetized with 5 mm sodium azide. Laser ablation was performed with a micropoint system running a dye‐pumped laser system with a nanosecond‐range pulse rate. Targeted cells were identified by the expression of fluorescent markers and were confirmed as ablated 48 h after surgery by the lack of fluorescence signals.

## Conflicts of interest

The authors declare that they have no conflicts of interest relating to this manuscript.

## Supporting information


**Fig. S1** A high throughput screen identifies multiple chemicals that extend lifespan.
**Fig. S2** NP1 modulates pumping through glutamatergic signalling.
**Fig. S3** NP1 acts through a pathway involving *eat‐4* and *gar‐3*.

**Table S1** Excel File of statistics and summaries from lifespan results.
