# Supplementary material for: Chemical activation of a food deprivation signal extends lifespan
Source: Aging Cell. 2016 May 24;15(5):832–41. doi: 10.1111/acel.12492 (PMC5013014; doi:10.1111/acel.12492)
Supplement: Supplementary file 1 — Fig. S1 A high throughput screen identifies multiple chemicals that extend lifespan. Fig. S2 NP1 modulates pumping through glutamatergic signalling. Fig. S3 NP1 acts through a pathway involving eat‐4 and gar‐3. [file ACEL-15-832-s001.doc]

**Supplementary Figures:**

**Figure S1:**

***
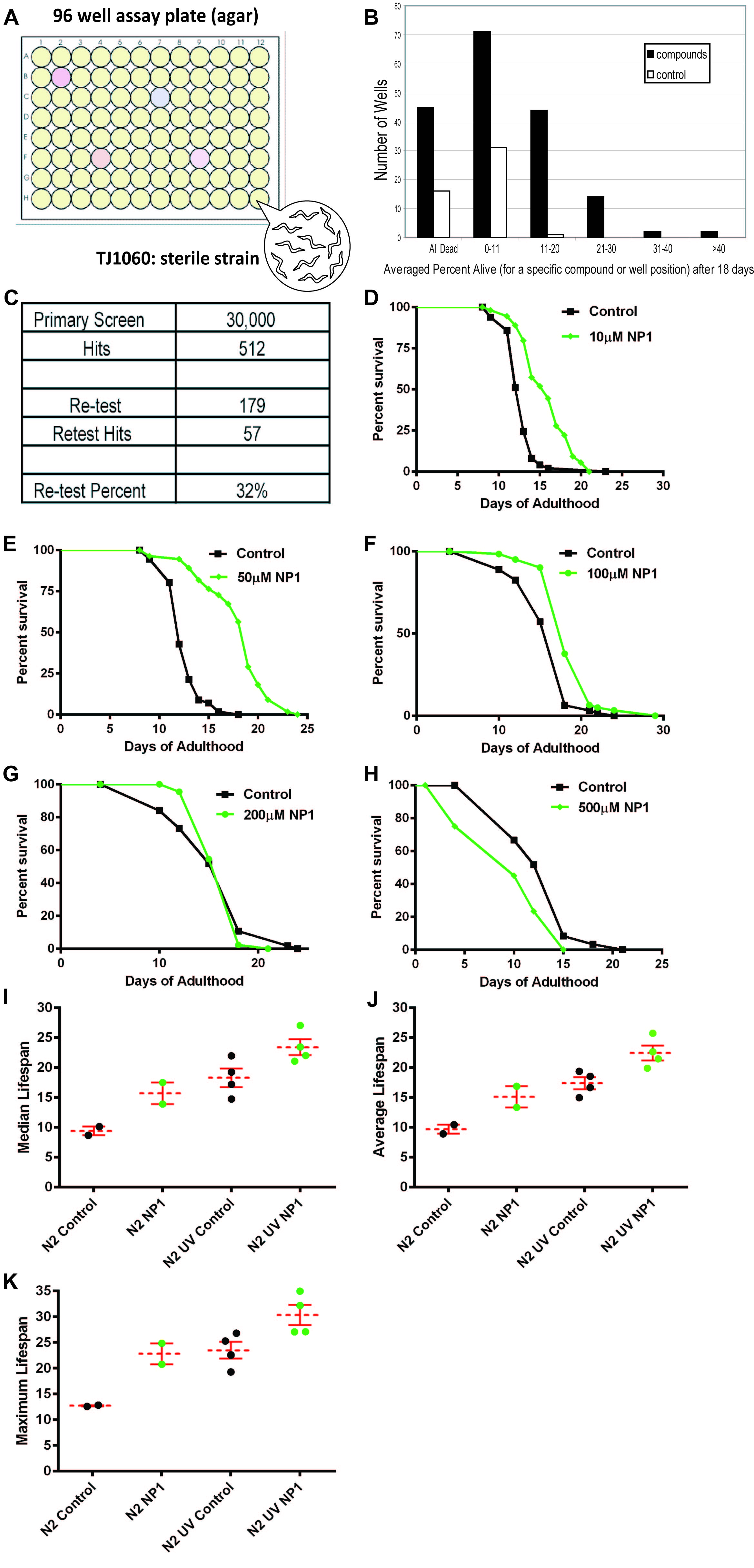
***

**Figure S1: *A high throughput screen identifies multiple chemicals that extend lifespan.***

A) A schematic describing the setup for the high throughput screen for chemicals that extend the lifespan of *C.elegans*. The screen utilized the strain TJ1060 which carries two temperature sensitive sterile mutations (*spe-9(hc88)I; fer-15(b26)II*) both of which are involved in sperm function. The screen was performed in a 96 well format with 150µL of NGM agar, concentrated OP50 and 10 worms in each well supplemented with DMSO control or 50µM chemical. (B) A graph of the binned results (control wells Vs. candidate chemicals) from the re-test experiment. The re-test was essentially a triplicate repeat of the primary screen (which was done in duplicate) using fresh chemical and shuffled well positions (only the inner 24 wells were used). The graphed results are biased in that the test wells out-numbered the control wells by 4:1 (representing averaged triplicate repeats of 2 plates for control and 8 plates for test). (C) Summary of the primary and re-test screens showing our call of 32% positives identified by the re-test (represents a cutoff of 11% alive which was the average of the control plus twice the standard deviation of the control). (D-H) Dose response of NP1 including results with varied vehicle concentrations. NP1 concentrations are described in the graphs. DMSO levels correspond to 0.05% in (D) 0.25% in (E) 0.5% in (F) 1% in (G) and 2.5% in (H). (I-K) Graphical results from an automated lifespan machine study, testing the lifespan extension effect of NP1 for a dependence on live bacteria. Results show scatter plots of the median (B), average (C) and maximum (longest lived animal) (D) lifespan for each replicate experiment. Middle bars show the average of the replicates. Outer bars show the standard error of the mean. See also Table S1.

**Figure S2:**

**
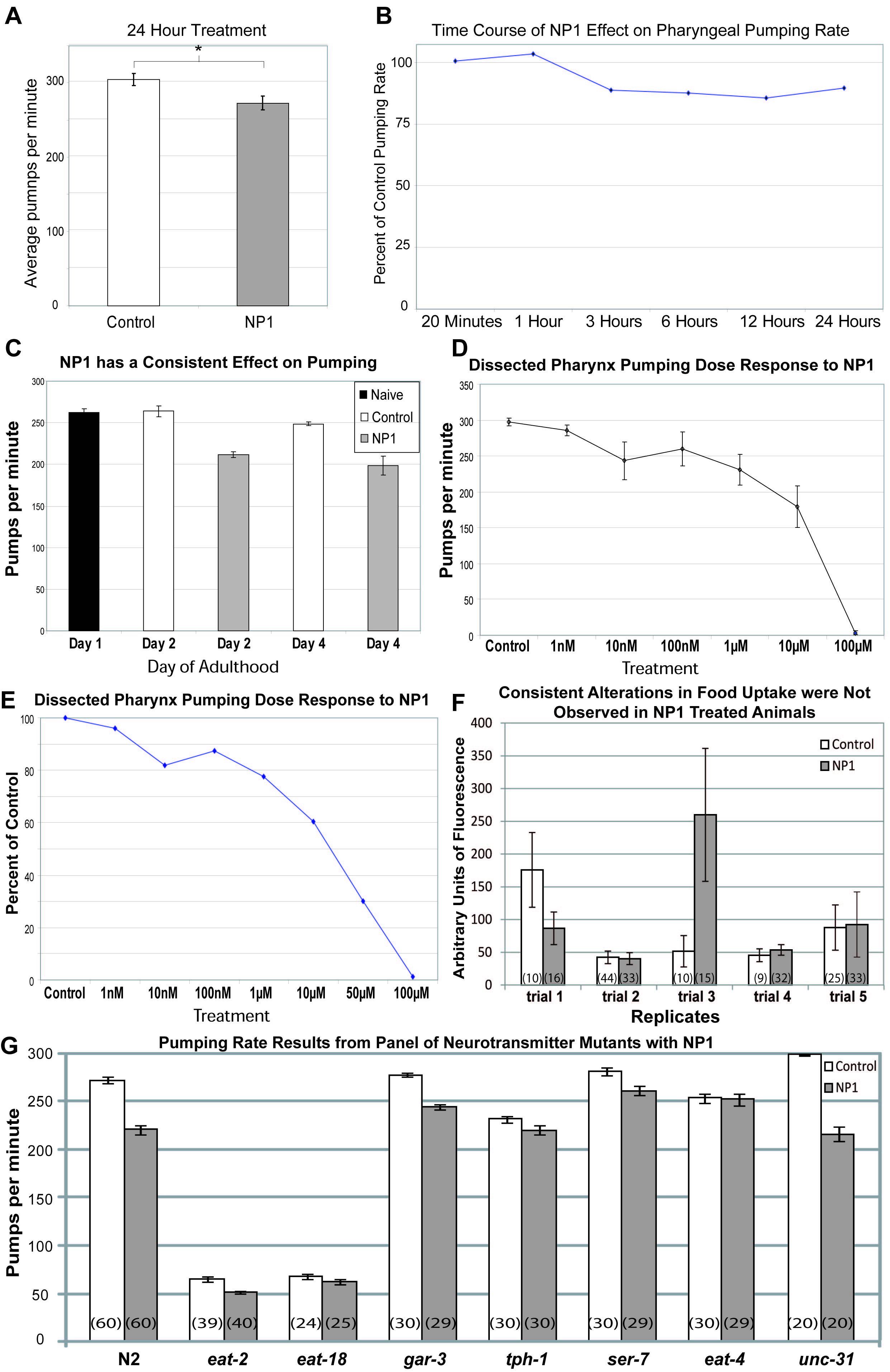
**

**Figure S2:** ***NP1 modulates pumping through glutamatergic signaling***

(A) NP1 caused a subtle but significant reduction in pumping rate. (B) A time course demonstrated that the NP1 effect on pumping rate peaked after 3 hours of incubation. (C) Treatment of wildtype animals with NP1 for 24 or 72 hours had a similar effect on pumping rate. Data in this graph was collected by manual scoring of pharyngeal pumps over 30s and was assayed in real time. (D-E) Dissected pharynxes were treated with control (DMSO) or varied concentrations of NP1. Assays were performed in the presence of 5-HT and pumping was manually counted over 30s. (D) A graphical representation showing pumps/min, while (E) includes an additional concentration and shows the effect on pumping relative to the control treatment. The 50µM concentration point is not included in (D) since it was performed at a lower 5-HT concentration than all other points (2µM instead of 20µM). (F) Shows the results from 5 independent trials testing for differences in GFP labeled OP50 accumulation after treatment with control or NP1. Adult day 1 worms were treated for 24 hours on regular food. Worms were then rinsed off and washed with M9 buffer 3X. Worms were placed on full plate lawns of GFP-OP50 to feed for 5 minutes. They were then rinsed off and washed 3X with M9 containing 100µM sodium azide and imaged. Images were analyzed in image J as previously described (Lucanic *et al.* 2013a). (G) Shows the pumping rates determined for the panel of neurotransmitter mutants described in main text (Figure 3C) and contains the averaged data used to generate figure 3C. Pumping rates in this figure were determined by manually counting pumps over 30s in real time. Parentheses in the bar graphs indicate the number of worms (n) averaged for that specific column. In all figures error bars represent the standard error of the mean. See also Table S1.

**Figure S3:**

**
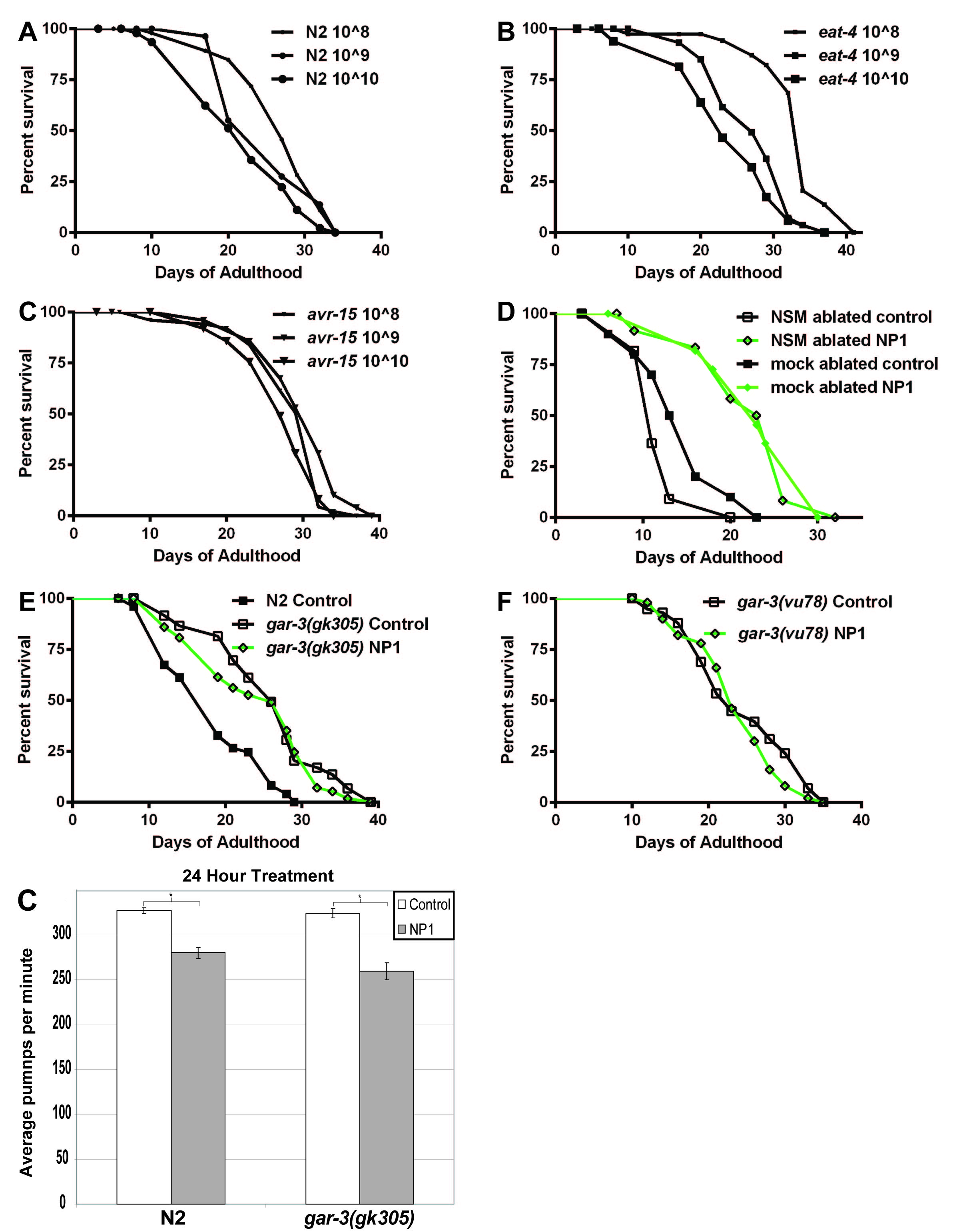
**

**Figure S3: *NP1 acts through a pathway involving eat-4 and gar-3***

(A-C) Wildtype and glutamatergic signaling mutants’ lifespans were modulated by the abundance of food. Wildtype (A), *eat-4(ky5)* (B) and *avr-15(ad1051)* (C) all respond to decreased food availability with lifespan extension. (D) Representative survivorship plot showing the lifespan of populations that either had their NSM neurons ablated with a laser or were mock ablated. *zdIs13[Ptph-1::GFP]* transgenic worms (used to identify the NSM neurons) responded to NP1 treatment with lifespan extension whether or not they possessed NSM neurons. (E-F) Multiple *gar-3* mutants (*gk305* and *vu78*) are long-lived relative to wildtype and do not respond to NP1 with further lifespan extension. The putative *gar-3* null allele *gk305* responds to NP1 with a decrease in pumping that is similar in magnitude to the wildtype response (G). Pumping rates shown in this figure were determined through video analysis of 10 s clips as described in the methods section. Error bars show the standard error of the mean. See also Table S1
