# Supplementary material for: Chemical activation of a food deprivation signal extends lifespan
Source: Aging Cell. 2016 May 24;15(5):832–41. doi: 10.1111/acel.12492 (PMC5013014; doi:10.1111/acel.12492)
Supplement: Supplementary file 2 — Table S1 Excel File of statistics and summaries from lifespan results. [file ACEL-15-832-s002.pdf]

experiment	median survival	mean lifespan	nt median lifespan change w	nt median survival different from w	deaths recorded (censored animals)	P value (logrank test)	Used in figure (red is supp.)
treatment from birth							
N2 Control	18	19			74		@
N2 NP1	25	25	39		97	P<0.0001*	@
treatment from birth							
N2 Control	20	20			93(6)		
N2 NP1	25	25	25		84(20)	P<0.0001*	
Blind from adult							
N2 control	19	20			57(5)		
N2 NP1	29	29	53		60(2)	P<0.0001*	
Blind from adult							
N2 control	19	18			53(7)		
N2 NP1	26	24	37		43(16)	0.0001*	
Blind from adult							
N2 control	23	21			55(3)		
N2 NP1	26	24	13		43(14)	0.0383*	
from adulthood							
N2 Control	12	13			56		
N2 NP1	19	18	58		55	P<0.0001*	
from adulthood							
N2 Control	15	16			54		
N2 NP1	21	21	40		57	P<0.0001*	
from adulthood							
N2 Control	13				58(2)		@
N2 NP1	27		108		62(1)	P<0.0001*	@
*compared to DMSO treated/same strain control							
dosage response							
N2 10μM Control	13	13			49		@
N2 10μM NP1	16	16	23		54	P<0.0001	@
N2 50μM Control	21	21			40		@
N2 50μM NP1	29	27	38		57	P<0.0001	@
N2 100μM Control	18	16			63		@
N2 100μM NP1	18	19	0		61	P<0.0001	@
N2 200μM Control	18	16			56		@
N2 200μM NP1	18	17	0		44	0.98	@
N2 500μM Control	15	13			60		@
N2 500μM NP1	10	10	-33		60	P<0.0001	@
Controls contain matching DMSO levels							
longevity pathways							
N2 Control	13	16			43(36)		
N2 NP1	27	25	108		75(6)	P<0.0001	
daf-16(mu86) Control	13	13		0	77(3)		
daf-16(mu86) NP1	16	15	23		75(5)	P<0.0001*	
N2 Control	15	16			70		@
N2 NP1	22	22	47		67	P<0.0001*	@
daf-16(mu86) Control	12	12		-20	66		@
daf-16(mu86) NP1	15	15	25		66	P<0.0001*	@
N2 control	17	18			59(5)		
N2 NP1	22	24	29		61(1)	P<0.0001*	
hsf-1(sy441) control	9	9		-47	42(17)		@
hsf-1(sy441) NP1	12	12	33		35(24)	P<0.0001*	@
RNAi (L4440) Control	20	21			43(17)		
RNAi (L4440) NP1	27	28	35		51(9)	P<0.0001*	
RNAi (*hsf-1)* Control	15	14		-25	43(21)		
RNAi *(hsf-1)* NP1	15	16	0		56(14)	0.0019*	
RNAi (L4440) Control	21	20			42(12)		
RNAi (L4440) NP1	29	27	38		52(6)	P<0.0001*	
RNAi (*hsf-1*) Control	13	14		-38	52(2)		
RNAi (*hsf-1*) NP1	18	18	38		64(2)	P<0.0001*	
RNAi (L4440) Control	20	21			43(17)		
RNAi (L4440) NP1	27	28	35		51(9)	P<0.0001*	
RNAi (*pha-4*) Control	25	24		25	55(15)	0.014#	
RNAi (*pha-4*) NP1	25	24	0		61(9)	0.3072*	
RNAi (*skn-1*) Control	20	20		0	50(20)		
RNAi (*skn-1*) NP1	22	22	10		66(1)	0.0173*	
RNAi (L4440) Control	21	20			42(12)		
RNAi (L4440) NP1	29	27	38		52(6)	P<0.0001*	
RNAi (*pha-4*) Control	21	23		0	53(2)	0.0982#	@
RNAi (*pha-4*) NP1	18	21	-14		57(7)	0.7905*	@
RNAi (*skn-1*) Control	21	19		0	55(5)		@
RNAi (*skn-1*) NP1	21	23	0		25(1)	0.0005*	@
*compared to DMSO treated/same strain control							
#comnpared to RNAi (L4440) Control							
N2 control	19	19			53		
N2 NP1	26	25	37		23	P<0.0001*	
osm-3(p802) control	33	30		74	35		@
osm-3(p802) NP1	35	35	6		28	0.0039*	@
N2 control	17	18			59(5)		
N2 NP1	22	24	29		61(1)	P<0.0001*	
osm-3(p802) control	30	29		76	26(38)		
osm-3(p802) NP1	33	32	10		33(30)	0.0308*	
N2 Control	15	15			57(3)		
N2 NP1	16	20	7		54(6)	P<0.0001*	
isp-1(qm150)	19	19		27	55(9)		@
isp-1(qm150) NP1	23	23	21		48(10)	P<0.0001*	@
*compared to DMSO treated/same strain control							
bacterial dilution							
Trial 1							
10^7 Control	19	19			56(2)		@
10^7 NP1	16	18	-16		54(6)	0.5029*	@
10^8 Control	21	22			58(1)		@
10^8 NP1	21	22	0		60(3)	0.9848*	@
10^9 Control	19	21			55(7)		@
10^9 NP1	22	22	16		54(3)	0.0501*	@
10^11 Control	16	14			53(1)		@
10^11 NP1	19	19	19		54(1)	P<0.0001*	@
Trial 2							
10^7 Control	21	20			53(7)		@
10^7 NP1	16	16	-24		56(4)	P<0.0001*	@
10^8 Control	21	20			57(4)		@
10^8 NP1	21	21	0		58(4)	0.0178*	@
10^9 Control	20	20			56(4)		@
10^9 NP1	24	23	20		51(9)	P<0.0001*	@

**10^11 Control**	**15**	**15**			**58(2)**		@
**10^11 NP1**	**20**	**18**	**33**		**57(4)**	**P<0.0001***	@
**Trial 3**							
**10^7 Control**	**22**	**23**			**37(19)**		@
**10^7 NP1**	**17**	**16**	**-23**		**22(35)**	**P<0.0001***	@
**10^8 Control**	**22**	**23**			**51(8)**		@
**10^8 NP1**	**20**	**20**	**-9**		**34(26)**	**0.0054***	@
**10^9 Control**	**20**	**19**			**44(12)**		@
**10^9 NP1**	**22**	**22**	**10**		**53(6)**	**P<0.0001***	@
**10^11 Control**	**15**	**15**			**45(15)**		@
**10^11 NP1**	**20**	**18**	**33**		**49(8)**	**P<0.0001***	@
**Trial 4**							
**10^7 Control**	**20**	**20**			**25(24)**		@
**10^7 NP1**	**15**	**15**	**-25**		**22(36)**	**P<0.0001***	@
**10^8 Control**	**24**	**23**			**46(11)**		@
**10^8 NP1**	**20**	**20**	**-17**		**41(18)**	**0.0004***	@
**10^9 Control**	**20**	**19**			**52(8)**		@
**10^9 NP1**	**24**	**24**	**20**		**45(14)**	**P<0.0001***	@
**10^11 Control**	**15**	**15**			**51(8)**		@
**10^11 NP1**	**20**	**18**	**33**		**53(7)**	**P<0.0001***	@
bacterial dilution assays with TJ1060 strain at 25˚C							all data combined for figure 2G
*compared to DMSO treated concentration control							
compared to 10^11 control (here considered *ad libitum*)							
glutamateric signaling							
**N2 Control**	**16**	**18**			**54(7)**		@
**N2 NP1**	**26**	**25**	**63**		**59(2)**	**P<0.0001***	@
***eat-4(ad819)* Control**	**15**	**13**			**30(30)**	**P<0.0001#**	@
***eat-4(ad819)* NP1**	**12**	**13**	**-20**		**27(33)**	**0.7959***	@
**N2 Control**	**lost**				lost		
**N2 NP1**	**lost**				lost		
***eat-4(ad819)* Control**	**17**	**16**			**51(8)**		
***eat-4(ad819)* NP1**	**15**	**14**	**-12**		**47(15)**	**0.0054***	
**N2 Control**	**19**	**19**			**95(3)**		@
**N2 NP1**	**27**	**27**	**42**		**83(5)**	**P<0.0001***	@
***avr-15(ad1051)* Control**	**19**	**18**			**89(4)**		@
***avr-15(ad1051)* NP1**	**19**	**18**	**0**		**89(9)**	**0.8721***	@
**N2 Control**	**15**	**17**			**75(3)**		
**N2 NP1**	**17**	**19**	**13**		**59(10)**	**0.0118***	
***avr-15(ad1051)* Control**	**17**	**17**			**65(14)**	**0.9935#**	
***avr-15(ad1051)* NP1**	**17**	**17**	**0**		**44(29)**	**0.4245***	
NP1 interactions with eat-2 and gar-3							
**N2 Control**	**19**	**18**			**49(6)**		@
**N2 NP1**	**19**	**19**	**0**		**61(2)**	**0.2671***	
***gar-3(gk305)* Control**	**26**	**26**		**37**	**59(4)**	**P<0.0001#**	@
***gar-3(gk305)* NP1**	**26**	**24**	**0**		**57(5)**	**0.3621***	@
***eat-2(ad1116)* Control**	**21**	**21**		**-19**	**54(4)**	**0.0106#**	@
***eat-2(ad1116)* NP1**	**26**	**26**	**24**		**68(1)**	**0.0018***	@
**N2 Control**	**17**	**17**			**59(2)**		@
**N2 NP1**	**20**	**22**	**18**		**55(7)**	**P<0.0001*#**	@
***gar-3(gk305)* Control**	**20**	**21**		**18**	**53(1)**	**0.0002#**	@
***gar-3(gk305)* NP1**	**21**	**23**	**5**		**56(1)**	**0.1497***	@
***eat-2(ad1116)* Control**	**17**	**18**			**46(1)**	**0.3362#**	
***eat-2(ad1116)* NP1**	**22**	**23**	**29**		**56(7)**	**P<0.0001***	
**N2 Control**	**16**	**16**			**58(3)**		
**N2 NP1**	**25**	**24**	**56**		**49(15)**	**P<0.0001***	
***eat-2(ad1116)* Control**	**20**	**20**		**25**	**78(12)**	**P<0.0001#**	
***eat-2(ad1116)* NP1**	**25**	**24**	**25**		**98(3)**	**P<0.0001***	
**N2 Control**	**14**	**16**			**32**		
**N2 NP1**	**23**	**23**	**64**		**23**	**0.0001***	
***gar-3(vu78)* Control**	**19**	**18**		**36**	**55**	**0.3873#**	
***gar-3(vu78)* NP1**	**21**	**19**	**11**		**56**	**0.1366***	
**N2 Control**	**24**	**22**			**32**		@
**N2 NP1**	**26**	**25**	**8**		**46**	**0.9066***	
***gar-3(vu78)* Control**	**23**	**24**		**-4**	**58**	**0.0772#**	@
***gar-3(vu78)* NP1**	**23**	**24**	**0**		**52**	**0.2088***	@
**N2 control**	**23**	**22**			**62(2)**		
**N2 NP1**	**28**	**24**	**22**		**30(0)**	**0.0383***	
***gar-3(vu78)* Control**	**23**	**24**		**0**	**55(3)**	**0.012#**	
***gar-3(vu78)* NP1**	**26**	**27**	**13**		**43(14)**	**0.2042***	
**N2 control**	**16**	**18**			**50(8)**		
**N2 NP1**	**26**	**25**	**63**		**46(14)**	**P<0.0001***	
***gar-3(vu78)* Control**	**23**	**24**		**44**	**59(3)**	**0.0002#**	
***gar-3(vu78)* NP1**	**28**	**25**	**22**		**42(18)**	**0.7637***	
*compared to DMSO treated/same strain control							
#compared to N2 control							
eat-4; gar-3 epistatic analysis							
**N2 Control**	**12**	**15**			**55(5)**		
**N2 NP1**	**26**	**24**	**117**		**59(1)**	**P<0.0001*#**	
***eat-4(ky5)* Control**	**16**	**15**		**33**	**49(22)**	**0.4275#**	@
***eat-4(ky5)* NP1**	**16**	**15**	**0**		**49(28)**	**0.2838***	
***gar-3(gk305)* Control**	**21**	**22**		**75**	**57(2)**	**P<0.0001#**	@
***gar-3(gk305)* NP1**	**16**	**19**	**-24**		**57(5)**	**0.0251***	6[illegible]
***eat-4; gar-3* Control**	**16**	**17**			**52(25)**	**0.1365%, P<0.0001$**	@
***eat-4; gar-3* NP1**	**16**	**16**	**0**		**35(29)**	**0.9105***	
**N2 Control**	**14**	**15**			**22(3)**		
**N2 NP1**	**23**	**21**	**64**		**35(23)**	**P<0.0001*#**	
***eat-4(ky5)* Control**	**16**	**17**		**14**	**33(25)**	**0.0392#**	
***eat-4(ky5)* NP1**	**14**	**14**	**-13**		**45(14)**	**0.1034***	
***gar-3(gk305)* Control**	**16**	**18**		**14**	**24(1)**	**0.0954#**	6[illegible]
***gar-3(gk305)* NP1**	**16**	**18**	**0**		**56(6)**	**0.8005***	6[illegible]
***eat-4; gar-3* Control**	**16**	**15**			**47(22)**	**0.0141%, 0.0201$**	
***eat-4; gar-3* NP1**	**16**	**17**	**0**		**49(20)**	**0.0406***	
*compared to same strain control							
#compared to N2 control							
% compared to eat-4(ky5) single							
$compared to gar-3(gk305) single							
laser ablations							
***zdIs13* mock ablated Control**	**15**	**14**			**10(2)**		@
***zdIs13* mock ablated NP1**	**23**	**24**	**59**		**11(1)**	**0.0005***	@
***zdIs13* NSM ablated Control**	**11**	**12**			**11(1)**		@
***zdIs13* NSM ablated NP1**	**25**	**23**	**123**		**12(1)**	**0.0001***	@
***zdIs13* mock ablated Control**	**17**	**17**			**10(0)**		
***zdIs13* mock ablated NP1**	**23**	**23**	**35**		**3(0)**	**0.0305***	
***zdIs13* NSM ablated Control**	**14**	**13**			**5(0)**		
***zdIs13* NSM ablated NP1**	**23**	**21**	**64**		**6(0)**	**0.0319***	

eat-4 NSM ectopic expression							
eat-4(ky5) non transgenic sibs Control	13	14			39(13)		
eat-4(ky5) non tansgenic sibs NP1	15	16	15		35(24)	0.0049*	@
eat-4;rfex228 Control	13	13			28(7)	0.988#	
eat-4; rfex228 NP1	24	21	85		47(22)	P<0.0001*	@
eat-4; rfex229 Control	13	14			51(12)	0.5203#	
eat-4; rfex229 NP1	19	20	46		48(15)	P<0.0001*	@
eat-4; rfex230 Control	13	12			21(10)	0.0478#	
eat-4; rfex230 NP1	19	19	46		25(4)	P<0.0001*	@
eat-4(ky5) non transgenic sibs Control	13	13			31(23)		@
eat-4(ky5) non tansgenic sibs NP1	19	18	46		32(17)	P<0.0001*	
eat-4;rfex228 Control	15	16			55(8)	0.0152#	@
eat-4; rfex228 NP1	17	17	13		56(7)	0.4411*	
eat-4; rfex229 Control	13	12			58(2)	0.197#	@
eat-4; rfex229 NP1	19	20	46		55(6)	P<0.0001*	
eat-4; rfex230 Control	10	11			18(6)	0.0013#	@
eat-4; rfex230 NP1	17	18	70		29(2)	P<0.0001*	
rfEx228-230[Ptph-1::eat-4]							
gar-3 tissue specific rescue							
gar-3(gk305) non transgenic sibs Control	22	22			58(4)		@
gar-3(gk305) non transgenic sibs NP1	20	20	-9		54(4)	0.4063*	@
gar-3; rfex224 Control	22	22			59(3)	0.5596#	@
gar-3; rfex224 NP1	27	27	23		63(1)	0.0002*	@
gar-3; rfex225 Control	20	20			51(7)	0.0306#	
gar-3; rfex225 NP1	20	22	0		63(3)	0.055*	
rfEx224-225[Pmyo-2::gar-3]							
*compared to same strain control							
#compared to NTS control							
glutmate mutant DR response							
N2 10^8	27	27			46(5)	0.0009*	@
N2 10^9	27	25			8(39)		@
N2 10^10	23	22			45(5)		@
eat-4(ky5) 10^8	34	32			17(31)	P<0.0001*	@
eat-4(ky5) 10^9	27	26			32(18)		@
eat-4(ky5) 10^10	23	23			37(15)		@
avr-15(ad1051) 10^8	32	28			47(5)	0.1356*	@
avr-15(ad1051) 10^9	32	30			49(2)	0.0019*	@
avr-15(ad1051) 10^10	27	27			49(2)		@
* compared to 10^10							
automated lifespan machine (ALM)							
test for dependence on live bacteria							
ALM UV NP1 individual plates							
N2 Control	10.1				27		@
N2 Control	8.7				25		@
N2 NP1	13.9				29		@
N2 NP1	17.5				12		@
N2 UV Control	17.2				27		@
N2 UV Control	22.0				17		@
N2 UV Control	14.7				19		@
N2 UV Control	19.2				26		@
N2 UV NP1	23.4				24		@
N2 UV NP1	22.0				23		@
N2 UV NP1	27.0				25		@
N2 UV NP1	21.1				28		@
ALM UV NP1 data combined plates							
N2 Control	9.4				52		@
N2 NP1	14.0		50		41	P<0.0001*#	@
N2 UV Control	17.5				89	P<0.0001#	@
N2 UV NP1	24.0		38		100	P<0.0001*	@
*compared to same strain control							
#compared to N2 control							
! ((Np1-Control)/(Control)*100							
